# Abdominal pregnancy with a healthy newborn: a new case

**DOI:** 10.11604/pamj.2019.34.35.20169

**Published:** 2019-09-16

**Authors:** Abderrahim Siati, Taher Berrada, Aziz baidada, Aicha Kharbach

**Affiliations:** 1 Department of Gynecology and Obstetrics, Souissi Maternity, Ibn Sina University Teaching Hospital, Mohamed V University, Rabat, Morocco

**Keywords:** Abdominal pregnancy, term, hemorrhage, laparotomy

## Abstract

Abdominal pregnancy is a rare form of ectopic pregnancy with very high morbidity and mortality for both the mother and the fetus. Diagnosis and management can pose some difficulties especially in low-resource centers. We report a case of abdominal pregnancy with a healthy newborn. A 34-year-old Moroccan woman, G4P3 (3 alive children), presented with shortness of breath and progressive abdominal distension and pain at 37 weeks' gestation. An emergency laparotomy was performed with the impression of abdominal pregnancy. Intraoperatively, the fetus was seen in an intact amniotic sac in her abdomen, the placenta was strongly adhered to the fundus and enveloped the left adnexa. A total hysterectomy with a left adnexectomy was performed. The patient and neonate progressed well and were discharged. Term abdominal pregnancy is an extremely rare diagnosis. The life-threatening complication is bleeding from the detached placental site. High index of suspicion is vital in making prompt diagnosis in such situations.

## Introduction

Abdominal pregnancy is a rare form of ectopic pregnancy with high morbidity and mortality for both the mother and the fetus. Ectopic pregnancy represents about 1-2% of all pregnancies, with 95% of those occurring in the fallopian tubes [1]. The incidence of abdominal pregnancy differs in various publications and ranges between 1:10,000 and 1:30,000 pregnancies [2]. Diagnosis can be frequently missed in most poor-resource settings because of poor antenatal coverage, low socioeconomic status in most of the patients as well as lack of adequate medical resources [3]. Advanced abdominal pregnancy is still rare, and guidelines for its management are yet unclear with few cases published to date in Africa [4]. We present a rare case of abdominal pregnancy at 37 weeks' gestation with a live baby without any malformation and good maternal outcome.

## Patient and observation

**Figure 1:**
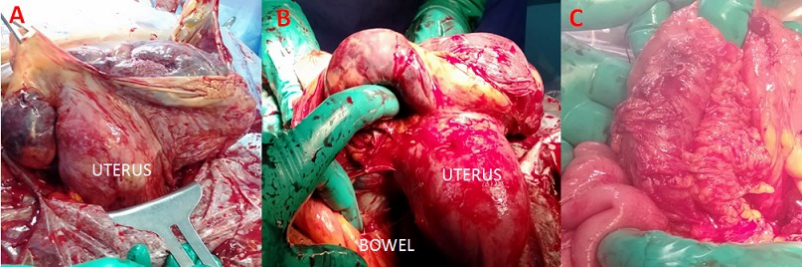
A) placenta location and uterus after delivery of the baby. The placenta was extensively adhered to the fundus and left adnexa; B) representing adherence of the placenta to the large bowel and fundus; C) the appearance of a segment of large bowel after the removal of placenta

**Figure 2:**
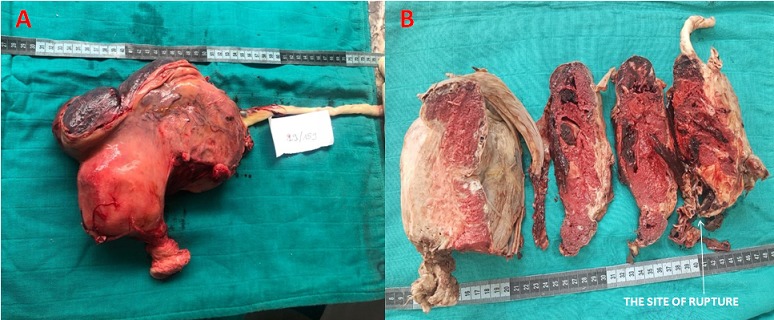
A) hysterectomy specimen with placenta invading the totality of the left adnexa; B) representing sections of the placenta related to the left fallopian tube. The site of rupture occurred at the interstitial portion of the fallopian tube, and that was confirmed with microscopic exam

A case of 34-year-old Moroccan woman who came from a rural area, G4P3 (3 alive children), presented to our emergency department at 37 weeks' gestation with a 3-day history of progressively worsening abdominal pain. The principal complaint on arrival was shortness of breath with associated progressive abdominal distension. She had no particular medical and surgical history. She was seen by a health professional only once during the current pregnancy at the regional hospital. Her last menstrual period was unknown but she reported 9 months of amenorrhea. On examination, she looked generally stable. She was not pale; vital signs were within normal parameters. On abdominal examination her symphysis fundal height was term sized, with longitudinal lie and breech presentation. The fetal heart rate was 139 beats per minute and there were no uterine contractions. On vaginal examination the cervix was closed and uneffaced. There was no vaginal bleeding. On ultrasonography examination, there was a singleton live pregnancy with a low quantity of amniotic fluid. The uterus was empty and the placenta appeared to be attached to the fundus. The gestational age was 37 weeks by ultrasound estimation. An emergency laparotomy was performed with the impression of abdominal pregnancy. A live female neonate was delivered weighing 2800g and the Apgar score was 6 and 8 in 1 and 5 min, respectively. No anomaly was seen on the baby. The fetus was seen in an intact amniotic sac and there was no hemoperitoneum. The placenta was strongly adhered to the fundus and the left adnexa (Figure 1 A). The uterus and the right adnexa were normal but the left fallopian tube was not identified. The amniotic sac was attached to segments of large bowel (Figure 1 B) and this was removed intraoperatively with no damage (Figure 1 C). A total hysterectomy with a left adnexectomy was performed and haemostasis was secured. Total estimated intraoperative blood loss was 1500ml. She was transfused with 4 units of packed red blood cells intra-and postoperative period. Both the mother and the baby progressed well, and were kept in the hospital for one week postoperatively, during which the mother recovered well from surgery, with no signs of infection or bleeding. They were discharged home, with a follow-up appointment in one month. The mother and child have been on regular follow up. All investigations by the neonatologist and the paediatrician did not show any abnormality on the baby. The histopathology report show an intra-abdominal pregnancy and the site of rupture was occurred at the interstitial portion of the fallopian tube (Figure 2). The placenta was at term and invading the totality of the left adnexa (Figure 2).

## Discussion

Abdominal pregnancy can be classified as primary or secondary. It is primitive in case of direct implantation of the embryo in the abdominal cavity; it is the less common type. It is secondary when it occurs after a ruptured tubal pregnancy or a tubal abortion or even a uterine rupture or perforation [5]. In our case the abdominal location is secondary because the embryo was first inserted into the interstitial portion of fallopian tube, as shown by the implantation of the placenta into this part of the tube. The diagnosis of abdominal pregnancy is difficult, and is an intra-operative finding in 40 to 50% of cases [5], despite antenatal follow-up and ultrasound scan. The clinical expression of abdominal pregnancy is variable, depending on the degree of the anatomical distortion it creates and the placental insertion site [6]. Clinical signs are therefore not specific: abdominal pain with intestinal transit disorder, abdominal pain during active movements of the fetus, spreading of the abdomen due to an irregular presentation, palpation of the fetal parts under the maternal abdominal wall [5,6]. Unfortunately most of these signs only appear during already advanced abdominal pregnancies, as with our patient. Once the condition is suspected, due to fetal malpresentation, malformations or oligohydramnios, then purposeful lateral projection sonography and radiography are helpful. An oxytocin stimulation test and the finding of an abnormally high maternal serum alfa-fetoprotein have been proposed [7]. Other radiological studies such as magnetic resonance imaging and computed tomography scan are helpful in the later stages. In our case the diagnosis was difficult and missed on initial ultrasound at the regional hospital. The treatment of abdominal pregnancy is surgical, at best by laparotomy, for a better control of the hemorrhagic risk related to the extraction of the placenta [5,8]. Bleeding from the placental site can be a lifethreatening complication during laparotomy. It is generally recommended to leave the placenta in situ and monitor the patient's human chorionic gonadotropin levels [2,9]. The use of methotrexate to accelerate the resorption is controversial for it would involve a greater risk of infection due to an accelerated placental necrosis [5,6]. When the placenta is left in place, it is necessary to keep watch over the appearance of the following maternal complications in post operative period: bowel obstruction, infection, hemorrhage, anemia, fistula, [10,11] etc. These complications can worsen the maternal prognosis, with a lethality up to 18% [5,7]. In this case, the placenta was strongly adhered to the fundus and the left adnexae, and there was significant bleeding from a detached portion of the placenta and the uterus, which required total abdominal hysterectomy with a left adnexectomy. For the newborn, it is very important to rule out congenital malformations. There are reports of fetal malformations as high as 40% associated with abdominal pregnancies [2]. When the diagnosis is late, or when it is done intra-operatively, the fetal prognosis is often very pessimistic, with a perinatal mortality which varies between 40% and 95% according to authors [5,6,10,11]. In this case no congenital malformations were detected and the baby showed normal morphological appearance and reflexes on examination.

## Conclusion

Abdominal pregnancy with a healthy newborn is an extremely rare situation. Its diagnosis is difficult so careful examination of the pregnant woman is important. The health authorities of our developing countries should make an effort to make routine early ultrasound accessible to pregnant women, and the obstetricians should keep in mind the possibility of ectopic pregnancy, irrespective of the gestational age.

## Competing interests

The authors declare no competing interests.
